# The potential roles of circRNAs in osteoarthritis: a coming journey to find a treasure

**DOI:** 10.1042/BSR20180542

**Published:** 2018-10-31

**Authors:** Hui-Zi Li, Zhong Lin, Xiang-He Xu, Nan Lin, Hua-Ding Lu

**Affiliations:** 1 Department of Orthopaedics, The Fifth Affiliated Hospital of Sun Yat-Sen University, Zhuhai, Guangdong Province 519000, China; 2 Center for Interventional Medicine, The Fifth Affiliated Hospital, Sun Yat-sen University, Zhuhai, Guangdong Province 519000, China; 3 Guangdong Provincial Engineering Research Center of Molecular Imaging, The Fifth Affiliated Hospital, Sun Yat-sen University, Zhuhai, Guangdong Province 519000, China

**Keywords:** biogenesis, CircRNA, Osteoarthritis

## Abstract

Osteoarthritis (OA), a common joint disease in elderly, causes serious social and economic burdens worldwide. Previous studies indicated that some differentially expressed circular RNAs (circRNAs) participated in the initiation and progression of OA. These findings suggested that circRNAs may act as promising diagnostic biomarkers and therapeutic targets for OA. In this review, we summarize the biogenesis and biological functions of circRNAs and explore the underlying roles of circRNAs in OA, which may enlighten further studies and contribute to the early diagnosis and intervention of OA.

## Introduction

Circular RNAs (circRNAs), a new type of RNAs originating from skipping of exons during alternative splicing of pre-mRNAs, extensively exist in organisms ranging from prokaryotes and eukaryotes to mammalians [1–3]. Unlike their linear counterparts, such as mRNAs and lncRNAs, circRNAs are characterized as covalently closed loop structure without 5′ caps and 3′ poly-A tails [4]. Also, the structure feature gives them the ability to resist the digestion of RNase R and RNA exonuclease, which enables them to stably express in organisms [4,5]. CircRNAs were once regarded as splicing errors without additional biological functions [6]. However, recent studies indicated that circRNAs can sponge miRNAs or functional proteins to regulate relevant biological functions at the transcriptional or post-transcriptional level [7,8]. Moreover, further studies revealed that circRNAs, such as Circ-FBXW7 and circ-SHPRH, can be translated into functional proteins with potential prognostic implications in cancer [9,10]. Therefore, circRNAs are not ‘splicing rubbish’, but a class of regulatory molecules with some important biological functions. Studies also indicated that dysfunction of circRNAs was associated with initiation and progression of several diseases, such as malignant tumors, atherosclerosis, degenerative diseases, and nervous system diseases [11–16]. Moreover, accumulated evidence demonstrated that circRNAs were promising diagnostic biomarkers and therapeutic targets for many diseases [17,18].

Osteoarthritis (OA), a common joint disease in the elderly, is associated with increasing medical burden in these years [19]. A recent study indicated that OA would pose a threat to the health of some 3.1 million people with relevant medical costs exceeding 2.9 billion Australian dollars until 2030 [20]. Persistent pain and progressive disability of affected joints are the main clinical symptoms of OA [21]. Generally, risk factors for OA are multi-factorial and complicated, which mainly include heredity, aging, gender, obesity, injury, and inflammation [22–25]. Considering the heterogeneity in etiology, symptoms, and prognosis among OA patients, some researchers suggested that more detailed-defined OA classification should be performed, which may contribute to individualized treatment for OA [26]. For instance, Herrero-Beaumont et al. [27] conducted an etiological classification of primary OA, including genetically determined OA, estrogen hormone-dependent OA, and aging-related OA. Also, Deveza et al. [28] classified OA patients into four phenotypes, which were mechanistic subgroups, pain subgroups, prognostic subgroups, and subgroups based on response to therapy. Some clinical guidelines recommended non-pharmacologic and pharmacologic therapies for early-stage OA, such as physical activities, oral and topical NSAIDs, and intra-articular injection treatments [29,30]. Regardless of the potential effectiveness in relieving symptoms, the pathological progress of OA can hardly be inhibited by following the aforementioned treatments and most of the OA patients still end up with joint replacement [31,32]. With the development of imaging techniques and biochemical markers, it is easier to diagnose OA than before. However, it is still hard to detect OA at an early stage [33]. The reason for this is that the key molecules and mechanisms switching on OA progression are still largely unclear. Therefore, it is essential for us to have a greater depth of understanding of OA pathogenesis. Recent studies found that numerous circRNAs were associated with OA, which suggested that they may play important roles in the initiation and progression of OA [34,35].

In this review, we will summarize the evidence on the biogenesis and biological functions of circRNAs and unveil potential mechanisms involved in OA. Furthermore, we will explore their possible clinical implications as early diagnostic biomarkers and therapeutic targets in OA.

## The discovery journey of circRNAs

**Figure 1:**
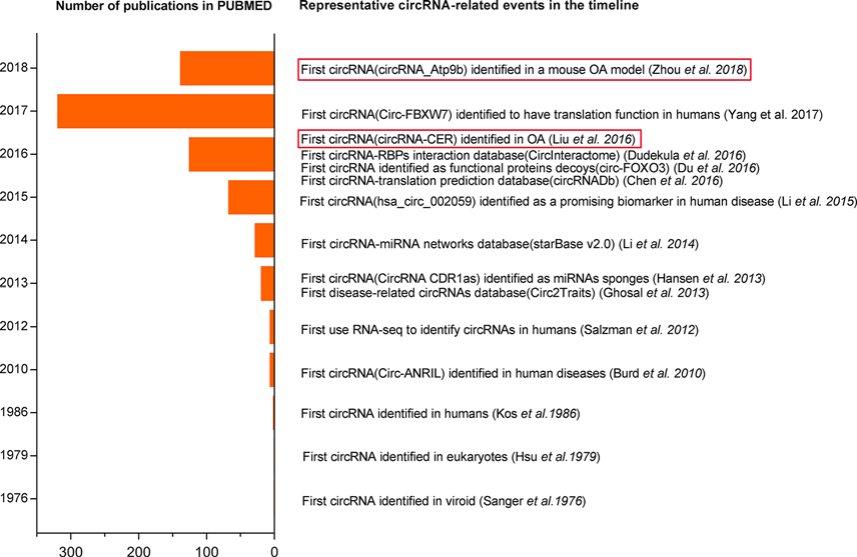
A timeline of representative events in circRNA research

CircRNAs were first discovered in plant-based viroids and eukaryotes using electron microscopy in 1970s [36,37]. And then, PCR amplification and sequencing verified the expression of circRNAs in humans in 1986 [38]. With the development of RNA-seq and bioinformatics, thousands of circRNAs were found in various species from virus, saccharomyces cerevisiae, mammals, to humans [39]. Although numerous circRNAs were found in humans, they were regarded as the splicing ‘by-products’ without additional biological functions [6]. Accidentally, Brud et al. [40] found that a novel non-polyadenylated circular RNA (circ-ANRIL) in 2010, which can resist RNAse R digestion, was associated with atherosclerosis risk, but the underlying pathogenesis related to circ-ANRIL in atherosclerosis was unclear. Since Hansen et al. [41] first found the ‘miRNA sponge’ function of ciRS-7 in 2013, researchers attached more attention to ‘the ancient molecules’. They demonstrated that circRNAs (CDR1as and Sry), like lncRNAs and mRNAs, were a class of important competitive endogenous RNAs (ceRNAs) with miRNAs response elements (MREs) and they can regulate the expression of target gene through binding to paired miRNAs [7]. From then, increasing studies verified that circRNAs had important biological functions including miRNA ‘sponge’, functional proteins decoys and translation function, and that the dysfunction of circRNAs was associated with the initiation and progression of various human diseases [8,9,42]. Interestingly, Liu et al. [35] first reported that 71 differentially expressed circRNAs participated in initiation and progression of OA in 2016; and of these, CircRNA-CER can act as a sponge of miR-136 to regulate the expression of MMP13, thus inducing extracelluar matrix (ECM) degradation. Subsequently, Zhou et al. [34] found that 255 circRNAs were differentially expressed in a mouse OA model; and of these, circRNA_Atp9b can promote ECM catabolism and inflammation in OA. The discovery journey of circRNAs is summarized in Figure 1.

## Biogenesis of circRNAs

**Figure 2:**
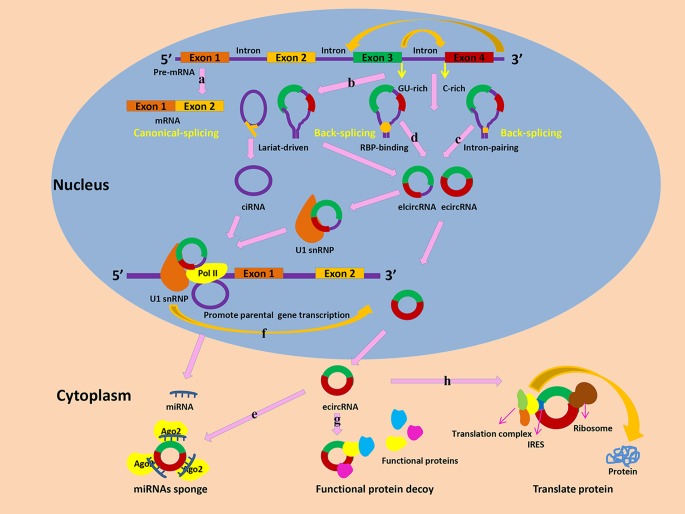
The biogenesis and biological functions of circRNAs (**A**) The canonical-splicing of pre-mRNA into mRNA. (**B**) Lariat-driven circularization. (**C**) Intron-pairing driven circularization. (**D**) RBP-binding driven circularization. (**E**) CircRNAs can act as miRNA sponges to inhibit miRNA function by forming miRNA-Ago2 complexes. (**F**) EIciRNAs and ciRNAs can interact with transcription complexes, such as U1 snRNP, to promote the transcription of their host genes. (**G**) CircRNAs can interact with functional proteins to affect relevant functions. (**H**) Translation function of circRNAs.

**Figure 3:**
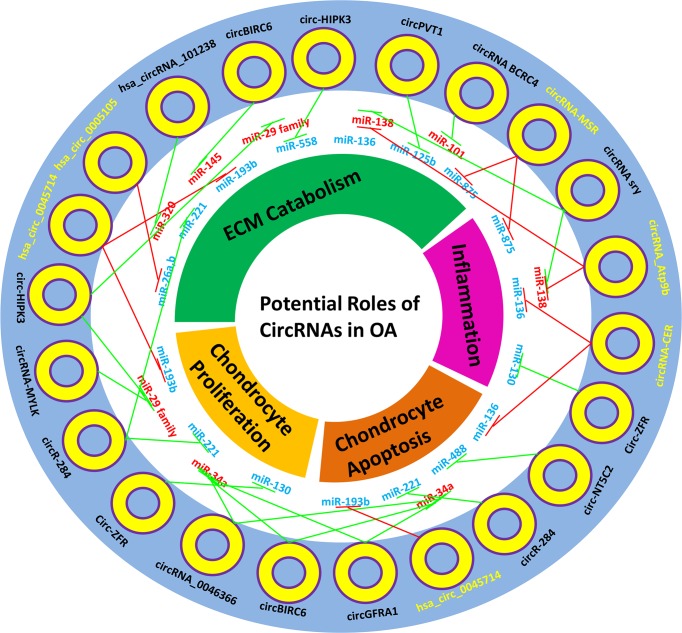
Potential ‘miRNAs sponges’ roles of circRNAs in OA Blue miRNAs denote down-regulated miRNAs in OA; Red miRNAs denote up-regulated miRNAs in OA; Yellow circRNAs denote experimentally-verified circRNAs in OA; Black circRNAs denote literature-supported circRNAs which may sponge OA-related miRNAs; Red edges denote experimentally-verified sponge relationships in OA; Green edges denote possible sponge relationships in OA.

According to their genomic origin and structure feature, circRNAs are divided into three subclasses: exonic circRNAs (ecircRNAs), intronic circRNAs (ciRNAs), and Exon-Intron circRNAs (EIciRNAs) [2]. Both circRNAs and mRNA originate from the alternative splicing of pre-mRNAs (Figure 2A) [43]. However, unlike the canonical splicing of pre-mRNA into mRNA, the special splicing process of circRNAs is called ‘the back-splicing’ that a downstream 3′ splice site is joined with an upstream 5′ splice site [4,44]. Circularization of circRNAs mainly includes lariat-driven circularization, intron-pairing driven circularization, and RNA-binding protein (RBPs) driven circularization. Lariat-driven circularization or exon skipping is an important model for circRNAs formation [45]. In the process of exon skipping, lariats with intron(s) and exon(s) are produced as the by-products of the canonical linear alternative splicing. And then, ecircRNA or EIciRNA is synthesized by the combination of 3′ tail of a downstream exon and the 5′ head of an upstream exon following the internal splicing of the lariats to remove intron(s) [46]. Also, the base pairing of intronic reverse complementary is important for the function of lariat driven circularization, which involves a seven-nucleotide GU-rich element located in the 5′ splice site and an 11-nucleotide C-rich element close to the branch point site (Figure 2B) [47]. Intron-pairing driven circularization is another important formation model of circRNAs [48,49]. The special ALU reverse complementary flanking sequences of intronic regions play critical roles in catalyzing the circularization (Figure 3B) [50,51]. Also, studies indicated that the special ALU sequences can maintain the balance between circRNAs and their linear counterparts, although the potential mechanism was unclear [51]. Similar to reverse complementary sequences in introns, some RBPs, such as QKI and MBL, also play vital roles in intron-pairing driven circulation model [44,52]. These RBPs with intronic binding sites interact with corresponding targeted introns to promote circularization (Figure 2D). A recent study indicated that the formation of ecircRNAs was triggered by insertion of the QKI sequence into linear RNA [53]. Besides the positive roles, RBPs also negatively regulate circularization. For instance, ADARs, a RBP with RNA-editing function, destroys the stem RNA structure to restrain the formation of circRNA. Furthermore, knockdown of ADARs promote the expression of circRNAs [47,54].

## Biological functions of circRNA

CircRNAs used to be regarded as splicing ‘rubbish’ without biological functions. However, increasing studies indicate that circRNAs extensively exist in eukaryotes and play important roles in the transcriptional, post-transcriptional, and translational levels. Generally, ecircRNAs are mainly located in cytoplasm and regulate post-transcriptional and translational levels, while ciRNAs or EIcircRNAs from nucleus have potential effects on transcription. Here, we summarize the biological functions of circRNAs.

## As a ceRNA or miRNA sponge

MiRNAs, a class of non-coding RNAs with some 20 nucleotides, bind to 3′ UTR of mRNAs to inhibit their translation [55]. Actually, the expression of mRNAs function orderly in organisms, which suggests that there may exist some potential mechanisms to suppress the functions of miRNAs. Of these, the competing endogenous RNA mechanism is a critical model to regulate the functions of miRNAs and balance gene expression [56]. Two studies first found that circRNAs with miRNA response elements acted as ‘miRNA sponges’ to regulate the expression of targeted genes in 2013 [7,41]. They documented that Cirs-7 was a circRNA containing more than 70 putative binding sites for miRNA-7 and it acted as a powerful miR-7 sponge to counteract its inhibited roles for targeted mRNAs (Figure 2E). Circular SRY, another famous circRNA, functions as a sponge for miR-138 to regulate the expression of targeted gene [41]. With the development of RNA-seq and bioinformatics, many new circRNAs are identified to contain MREs and act as ‘miRNAs sponges’, which further enrich the complexity of ceRNAs [57].

## Protein-binding functions

Beside miRNAs, circRNAs also bind to proteins to modulate the corresponding functions. The ciRNAs and EIcircRNAs, which are mainly located in the nucleus, do not function as ceRNAs, but regulate gene transcription through directly interacting with RBPs (Figure 2F) [58]. For instance, Ci-ankrd52 is found to richly distribute in transcription initiation site of the relevant gene and it can bind to RNA polymerase II to promote the transcription of the parental gene [59]. Knockdown of Ci-ankrd52 inhibits the expression of the linear mRNA ankrd52, but not its upstream or downstream genes. Two EIciRNAs, EIciEIF3J, and EIciPAIP2 can interact with U1 snRNP and Pol II to facilitate transcription of the host genes [58]. The circRNAs located in cytoplasm act as a decoy of functional proteins (Figure 2G). For example, circ-Foxo3 acts as a decoy of several cellular stress protein factors, such as the antisenescent protein ID-1, the transcription factor E2F1, the antistress proteins FAK, and HIF1alpha, to regulate senescence of mouse embryonic fibroblasts [60]. Also, circ-Foxo3 binds to CDK2 and P21 to develop a ternary complex, subsequently inhibiting cell cycle progression [8].

## Translation function

CircRNAs were used to be regarded as a class of non-coding RNAs. However, some facts always remind us of their potential translation function. For instance, most of circRNAs originate from protein-coding genes and many circRNAs located in the cytoplasm contain the translation start codon and open reading frames according to the CSCD database [61]. Previous studies have found that functional proteins are produced following by transfecting engineered circRNAs with internal ribosome entry sites, which is necessary for circRNA translation [62]. A recent study by Legnini et al. [63] have showed that Circ-ZNF609 is translated into a functional protein to regulate myoblast proliferation. Also, Pamudurti et al. [64] found that CircMbl3, a special circRNA from fly heads, is translated into a novel protein in a cap-independent way. More interestingly, Zhang and colleagues have demonstrated that cricRNAs are translated into functional proteins in humans for the first time. They show that circ-FBXW7 is translated into a novel 21-kDa protein, FBXW7-185aa, which is positively linked with overall survival of glioblastoma patients [9]. Also, they verify that SHPRH-146aa, a 17-kDa protein from circ-SHPRH, acts as a tumor suppressor in human glioblastoma (Figure 2H) [10]. Just consistent with the above prediction, are those circRNAs with translation functions originated from protein-coding genes, and contain the translation start codon and open reading frame. All of them are translated into functional protein using a splicing-dependent and cap-independent model.

## CircRNAs in OA: a coming journey to find a treasure

### Potential roles of circRNAs in OA

Generally, some intracellular and extracellular stress from OA-associated risk factors activates relevant signaling pathways and transcriptional factors, thereby resulting in the dysfunction of chondrocytes and the imbalance of extracellular matrix homeostasis. Previous studies indicated that circRNAs were differentially expressed at different pathological status of OA. Liu et al. [35] found that 71 circRNAs were aberrantly expressed in articular cartilage of OA patients. Of these, circRNA-CER was obviously up-regulated and silencing of circRNA-CER inhibited MMP13 expression and promoted ECM formation. They also found that 104 differentially expressed circRNAs were associated with mechanical stress-induced OA and silencing of circRNA-MSR inhibited TNF-α expression and increased ECM formation [65]. Zhou et al. [34] demonstrated that a total of 255 circRNAs were differentially expressed in a IL-1β-treated OA mouse model and knockdown of circRNA_Atp9b increased the expression of ECM and inhibited the release of inflammatory factors, such as COX-2 and IL-6 [66]. Actually, the exact mechanisms of these circRNAs in OA cartilage are largely unclear, but it is speculated that they are associated with stress and play important regulatory roles in pathogenesis of OA. It is plausible that some circRNAs may be stimulated to prevent the pathological development or dysregulated circRNAs aggravate the progression of OA [67]. Increasing evidences have suggested that circRNAs take a part in human diseases via ‘miRNA sponge’. Until now, only sporadic studies have reported the special roles of circRNAs in OA, with the similar conclusion that some circRNAs, act as miRNAs ‘sponge’ to modulate the expression of targeted mRNA in OA (Table 1) [34,35,65,66,68,69]. Actually, previous studies have revealed that ectopically expressed miRNAs from chronic stress, such as miR-29 family (a,b,c), miR-558, and miR-221 are associated with the initiation and progression of OA [70–72]. Moreover, several studies have indicated that some circRNAs, such as circ-HIPK3 and circRNA-284 can act as ‘sponges’ of these stress-related miRNAs to regulate the expression of targeted genes [73,74]. Therefore, it is essential to explore the potential roles of these circRNAs in OA, which may provide novel evidence of the ‘miRNA sponge’ mechanism in OA (Figure 3). On the other hand, is ‘sponge’ the only way to regulate progression of OA for circRNAs? Obviously, the answer is ‘No!’. Judging from the discovery history of circRNA functions, we track back to 2013, when Hansen et al. first identified the ‘sponge’ function of ciRS-7. Subsequently, their biogenesis and other regulatory functions, such as decoys of functional proteins and translation function, are gradually recognized. Moreover, RNA-seq and bioinformatics analysis largely promotes the understanding of circRNAs in human diseases. Thus, we can forecast that future studies will unveil more regulatory roles of circRNAs in OA, considering that the first study about circRNAs in OA is merely published in 2016. Anyway, these previous studies provide advantageous implications to enrich our understanding of circRNAs’ roles in OA.

**Table 1:** The expression of circRNAs in OA

Author	Year	Design	Main findings	References (PMID)
Liu et al. [35]	2016	Microarray; Bioinformatics analysis; qRT-PCR	A total of 71 circRNAs were aberrantly expressed in articular cartilage of OA patients. Of these, circRNA-CER was obviously up-regulated and silencing of circRNA-CER can inhibit MMP13 expression and promote ECM formation.	26931159
Liu et al. [65]	2017	Microarray; Bioinformatics analysis; qRT-PCR	A total of 104 differentially expressed circRNAs were identified in damaged versus intact cartilage. CircRNAs-MSR participated in TNF-α expression and was associated with cartilage matrix degradation.	28624198
Li et al. [69]	2017	qRT-PCR	Hsa_circ_0045714-miR-193b-IGF1R axis played an important role in extracellular matrix synthesis and chondrocytes proliferation and apoptosis.	28795385
Wu et al. [68]	2017	qRT-PCR	Hsa_circ_0005105-miR-26a-NAMPT axis played an important role in cartilage matrix degradation and expression of inflammation factors.	28276108
Zhou et al. [34]	2018	CircRNA sequencing; Bioinformatics analysis; qRT-PCR	A total of 255 circRNAs were identified to be differentially expressed in IL-1β-treated chondrocytes.	29247798
Zhou et al. [66]	2018	qRT-PCR	Knockdown of circRNA_Atp9b increased the expression of ECM and inhibited the release of such inflammatory factors as COX-2 and IL-6.	29305974

### CircRNA: a promising biomarkers in OA

The covalently closed ring structure of circRNAs gives them the ability to endure the degradation of RNase, which makes them express stably in the body. A recent study has found that thousands of circRNAs exist in human peripheral whole blood and some circRNAs have higher abundance than their linear counterparts, which suggests the underlying roles of circRNAs as diagnostic and prognostic biomarkers in the easily accessible body fluid [75]. Li et al. [76] have suggested that hsa-circRNA11783-2 in peripheral blood acts as a useful diagnostic biomarker for T2DM patients with coronary artery disease. Furthermore, the study by Zhao et al. [77] indicates that 22 circRNAs are differentially expressed in the peripheral blood of coronary artery disease patients and of these, hsa_circ_0124644 with the largest AUC is a promising diagnostic biomarker for coronary artery disease. Additionally, many aberrantly-expressed circRNAs are identified as the sensitive prognosis biomarkers in cancers. For instance, Xia et al. [78] have found that hsa_circ_0067934, a significantly up-regulated circRNA, is associated with poor differentiation, I-II T stage, and I-II TNM stage in esophageal squamous cell carcinoma. Han et al. [42] have demonstrated that low circMTO1 expression is related to poor prognosis in HCC patients. A recent finding by Jiang et al. [79] suggested that Cdr1as was up-regulated in cholangiocarcinoma and the overexpression of Cdr1as was linked to advanced TNM stage, lymph node invasion, postoperative recurrence, and worst overall survival. The conventional diagnostic methods for OA are based on imageology examination (i.e. X-ray and MRI) and clinical symptoms. However, these available methods are merely effective for detection of advanced OA, while they hardly recognize OA in earlier stage. Given that dysfunction of circRNAs is associated with the onset and progression of OA, circRNAs may play potential roles as early diagnostic and prognostic biomarkers in OA. There are no available studies to verify differentially expressed circRNAs in the accessible body fluids of OA patients, so further studies should be warranted to explore the potential diagnostic and prognostic value of circRNAs in OA.

### The therapeutic potentials of circRNAs in OA

Apart from potentials as biomarkers, circRNAs may act as promising therapeutic targets in OA. Zhang et al. [80] have suggested that circRNA_100269 is down-regulated and its targeted miRNA, miR-630, is up-regulated in gastric cancer. Furthermore, cell proliferation of gastric cancer cells is obviously inhibited following overexpression of circRNA_100269. Also, Li et al. [81] found that the expression of circ-104916 was down-regulated in gastric cancer and up-regulating circ-104916 inhibited the proliferation, migration, and invasion of gastric cancer cells. Additionally, a recent study has indicated that the treatment of hsa_circ_0045714 inhibits the progression of OA through promoting the expression of type II collagen and aggrecan, and chondrocyte proliferation [69]. On the other hand, numerous studies suggested that the dysfunction of miRNAs played vital roles in the onset and progression of OA [82]. The traditional methods to inhibit miRNA function contain gene knockout, antisense oligonucleotides, and miRNA sponges [83,84]. The construction of animal knockout models may spend much time and money, and the miRNA inhibitor function of antisense oligonucleotides may work well in short-term experiment, but not in long-term experiment [84,85]. Interestingly, miRNA sponges have similar miRNA-inhibited ability to antisense oligonucleotides *in vitro* [83,86]. More importantly, circRNA, a ‘super sponge’ with several MREs, may have more powerful ability to bind to targeted miRNAs when compared with its linear counterparts, such as mRNA and lncRNA. Jost et al. [87] investigated that artificial circRNAs sponges can down-regulate the level of miR-122, thus regulating the expression of corresponding proteins in a HCV model system. Therefore, artificial regulation of circRNAs may act as a promising gene therapy against functions of miRNAs to prevent the development of OA.

### CircRNAs in other joint diseases

As with OA, dysfunction of circRNAs is associated with the pathogenesis of other joint diseases, such as rheumatoid arthritis (RA) and intervertebral disc degeneration (IDD). Ouyang et al. [88] found that five circRNAs (092516, 003524, 103047, 104871, and 101873) were significantly unregulated in peripheral blood mononuclear cells from RA patients. Of these, circRNA_104871 could function as a potential diagnostical biomarker for RA. Zheng et al. [89] performed microarray analysis for peripheral blood mononuclear cells of 10 RA patients, which indicated that 255 circRNAs were significantly up-regulated and 329 down-regulated in the RA samples. Recent studies also indicate that circRNAs are also involved in development of IDD [90]. Cheng et al. [91] reveal that the level of CircVMA21 is significantly down-regulated in cytokines-treated nucleus pulposus (NP) cells and degenerative NP tissues. Furthermore, overexpression of CircVMA21 can alleviate the progression of IDD through miR-200c-XIAP axis *in vivo* and *in vitro*. The study performed by Guo et al. [15] indicates that circ-GRB10 can activate ERBB2 signaling pathway via sequestering miR-328-5p, thus inhibiting NP cell apoptosis and promote cell proliferation *in vitro*. Therefore, circ-GRB10 may act as novel diagnostic biomarkers and therapeutic targets for IDD. Actually, circRNA-related studies in non-OA joint diseases are still scarce, so further studies are necessary to explore the regulatory roles of circRNAs in these diseases.

## Conclusion and perspectives

Until now, only six studies explored the potential roles of circRNAs in OA. All of these studies suggested that the circRNAs–miRNAs–mRNAs axis played important roles in the pathogenesis of OA. However, we should be alert that the ‘sponge’ mechanism may be a potential pitfall in the pathogenesis of diseases. For instance, Li et al. [69] found that hsa_circ_0045714-miR-193b-IGF1R axis played an important role in extracellular matrix synthesis and chondrocytes proliferation and apoptosis. However, the expression level of hsa_circ_0045714 is down-regulated, while miR-193b up-regulated in OA. It is questionable to the ‘sponge’ role of hsa_circ_0045714 for miR-193b *in vivo*. Consequently, it is worthwhile to explore the potential roles of some low-abundance circRNAs in pathogenesis of diseases, when considering that they could hardly affect the functions of predicted miRNAs with higher abundance. In theory, at least seven binding sites are necessary for circRNA to ‘sponge’ miRNA. Therefore, it is questionable for some studies which verified the ‘sponge’ function *in vitro*, but with less than seven circRNA–miRNA binding sites from bioinformatics prediction. For example, Wu et al. [68] demonstrated that hsa_circ_0005105 acted as a sponge of miR-26a to promote extracellular matrix degradation in OA. However, only three consecutive binding sites were predicted between hsa_circ_0005105 and miR-26a in the study, so the ‘sponge’ relationship was needed for further experimental verification. The current available studies have merely unveiled the potential roles of OA-related circRNAs in ECM degradation, chondrocytes proliferation, apoptosis, and inflammation. Actually, previous studies indicate that differentially expressed miRNAs are associated with dysfunction of chondrocytes autophagy, oxidative stress and signaling pathways, which are correlated with pathogenesis of OA [92]. Therefore, it is essential to elucidate whether circRNAs also participated in these pathophysiological processes in OA. Apart from ‘sponge’ functions for downstream miRNAs, some circRNAs, such as circRNA FOXO3, are verified to act as decoys of several functional proteins to regulate relevant functions in human diseases [8,60]. Currently, there is no study focused on circRNA–protein interactions in OA. Some experimental methods, such as RNA pull-down assay, RNA immunoprecipitation, RNase protection assay, and fluorescence *in situ* hybridization techniques, are useful for exploring this novel mechanism in OA [93]. Moreover, recent studies indicated that some circRNAs, such as Circ-FBXW7 and circ-SHPRH, can be translated into functional protein, which participated in the development and progression of diseases [9,10]. The proteins translated from circRNA may be relatively non-functional under physiological conditions. However, intracellular and extracellular stress may promote the activation of the cap-independent translation model, so proteins encoded by circRNAs may play critical roles in pathological conditions [94]. Anyway, the special cap-independent translation mechanism of circRNAs is still largely unclear, which needs to be further investigated. Also, the underlying translation function of circRNAs in OA is worth exploring in future studies. One the other hand, the development of RNA-seq and bioinformatics promotes construction of such circRNA-related databases as circBase, circRNABase, RegRNA 2.0, Circ2Traits, circinteractome, circnet, deepbase version 2.0, circRNADb, CSCD, and circlncRNAnet, which contributes to further clarify the potential roles of circRNAs in OA (Supplementary Table S1) [61,95–103]. CircRNAs may be identified as promising diagnostic biomarkers and therapeutic targets in OA, but relevant studies are limited until now. Thus, further studies should be warranted to explore the roles of circRNAs in accessible body fluid of OA patients, such as peripheral blood and saliva, which may act as novel diagnostic biomarkers and therapeutic targets for OA.

In conclusion, although the circRNA-related studies in OA and other joint diseases are limited, these findings indicate that circRNAs participate in the pathogenesis of OA. Therefore, further studies should be performed to clarify the potential roles of circrRNAs in OA, which may contribute to the early diagnosis and intervention for OA.

## Supporting information

**Table S1:** CircRNA-related databases and their useful functions

## Abbreviations

ceRNA: competitive endogenous RNA
circRNA: circular RNA
ECM: extracelluar matrix
IDD: intervertebral disc degeneration
MRE: miRNAs response element
NP: nucleus pulposus
OA: osteoarthritis
RA: rheumatoid arthritis
RBP: RNA-binding protein
