# Supplementary material for: The potential roles of circRNAs in osteoarthritis: a coming journey to find a treasure
Source: Biosci Rep. 2018 Oct 31;38(5):BSR20180542. doi: 10.1042/BSR20180542 (PMC6209588; doi:10.1042/BSR20180542)
Supplement: Supplementary file 1 [file bsr20180542_Supp1.pdf]

**Table S1: CircRNA-related databases and their useful functions**

| Databases | Year | Function | Website |
|---|---|---|---|
| RegRNA 2.0 | 2013 | Predict miRNA-circRNA interactions | http://regrna2.mbc.nctu.edu.tw |
| Circ2Traits | 2013 | Collect disease-associated circRNAs | http://gyanxet-beta.com/circdb/ |
| circBase | 2014 | Provide gene description, genomic position of circRNAs | http://www.circbase.org |
| circRNABase | 2014 | Predict miRNA-circRNA interactions | http://starbase.sysu.edu.cn/mirCircRNA.php |
| circinteractome | 2016 | Predict miRNA-circRNA interactions;<br>Design junction-spanning primers of circRNAs;<br>Design siRNAs for circRNA silencing;<br>Dentify potential internal ribosomal entry sites (IRES) | http://circinteractome.nia.nih.gov |
| CircNet | 2016 | Provide tissue-specific circRNA expression profiles;<br>Provide circRNA-miRNA-gene regulatory networks | http://circnet.mbc.nctu.edu.tw/ |
| deepbase v2.0 | 2016 | Predict protein-circRNA co-expression networks | http://biocenter.sysu.edu.cn/deepBase/ |
| circRNADb | 2016 | Predict protein-coding function of circRNAs | http://reprod.njmu.edu.cn/circrnadb |
| CSCD | 2018 | Predict the microRNA response element sites of circRNAs;<br>Predict RNA binding protein sites of circRNAs;<br>Predict potential open reading frames of circRNAs | http://gb.whu.edu.cn/CSCD |
| circlncRNAnet | 2018 | Analyze expression profiles, co-expression networks and pathways, and molecular interactomes of circRNAs according to user-defined criteria. | http://app.cgu.edu.tw/circlnc/ |
